# Polymer models of topological insulators

**DOI:** 10.1186/1756-8935-6-S1-P127

**Published:** 2013-04-08

**Authors:** Boryana Doyle, Maxim Imakaev, Geoffrey Fudenberg, Leonid Mirny

**Affiliations:** 1 MIT-PRIMES, Massachusetts Institute of Technology, Cambridge, MA, 02139, USA; 2 Department of Physics, Massachusetts Institute of Technology, Cambridge, MA, 02139, USA; 3 Program in Biophysics, Harvard University, Boston, MA, 02115, USA; 4 Harvard-MITHealth Sciences and Technology, Massachusetts Institute of Technology, Cambridge, MA, 02139, USA

## Background

The classic model of eukaryotic gene expression requires spatial contact between a distal enhancer and a proximal promoter. To control gene expression, various models of enhancer-blocking insulators have been proposed, including a decoy model and a topological model [1]. The decoy model suggests that the enhancer or the promoter interacts with an insulating element to prevent enhancer-promoter interactions. The topological model suggests that two or more insulating elements interact with each other to form loops. An outstanding question in the field is whether the topological model is effective at preventing enhancer-promoter interactions.

## Materials and methods

Here we use the polymer model of chromatinized DNA and simulations of Brownian polymer dynamics to study the topological model of enhancer-blocking insulators. We consider one- and two-loop topological elements and assess spatial contacts between various regions of DNA induced or suppressed by an topological elements.

## Results

We find that a loop formed in the region between an enhancer and a promoter in fact facilitates enhancer-promoter contacts and does not act as an enhancer-blocking insulator. However, we find that sequestration of an enhancer (or promoter) within a loop is a plausible mechanism for topological insulators. Both the facilitating and insulating effects of topological elements are more dramatic with two-loop elements.

## Conclusions

Our polymer simulations demonstrate that the loop-forming topological elements are capable of both facilitating and insulating enhancer-promoter interactions. We note that the biological model of insulator activity around H19 and Igf2 genes in mouse liver cells presented by Kurukuti et al. [2] is a specific realization of the two-loop topological insulator discussed here. Our simulations show that the polymeric nature of chromatin is essential for modulating the action of topological insulators that modulate contact frequency between promoters and enhancers.
